# Natural Killer Cell Dysfunction in Obese Patients with Breast Cancer: A Review of a Triad and Its Implications

**DOI:** 10.1155/2021/9972927

**Published:** 2021-06-07

**Authors:** Esraa Elaraby, Abdullah Imadeddin Malek, Hanan W. Abdullah, Noha Mousaad Elemam, Maha Saber-Ayad, Iman M. Talaat

**Affiliations:** ^1^College of Medicine, University of Sharjah, Sharjah, UAE; ^2^Sharjah Institute for Medical Research, University of Sharjah, Sharjah, UAE; ^3^Faculty of Medicine, Cairo University, Cairo, Egypt; ^4^Faculty of Medicine, Alexandria University, Alexandria, Egypt

## Abstract

Natural killer cells (NK cells) are a crucial constituent of the innate immune system as they mediate immunity against viruses, bacteria, parasites, and most importantly, tumor cells. The exact mechanism of how the innate immune system and specifically NK cells interact with cancer cells is complex and is yet to be understood. Several factors that constitute the tumor microenvironment (TME) such as hypoxia and TGF-*β* are believed to play a role in the complex physiological reaction of NK cells to tumor cells. On the other hand, several risk factors are implicated in the development and progression of breast cancer, most importantly: obesity. Cytokines released from adipose tissue such as adipokines, leptin, and resistin, among others, are also believed to facilitate tumor progression. In this study, we aimed to build a triad of breast cancer, obesity, and NK cell dysfunction to elucidate a link between these pillars on a cellular level. Directing efforts towards solidifying the link between these factors will help in designing a targeted immunotherapy with a low side-effect profile that can revolutionize breast cancer treatment and improve survival in obese patients.

## 1. Breast Cancer and Its Microenvironment

Breast cancer is considered the most common type of cancer among women. The current global burden of breast cancer is substantial as it affected approximately 2.3 million women in 2020 alone. Moreover, breast cancer accounts for 1 in every 4 cancer cases among females and is the preeminent cause of death in women. It is estimated that 1 in 6 cancer deaths is due to breast cancer and the approximate number of deaths in 2020 was 684,996 [1]. Moreover, a wide range of risk factors related to breast cancer are reported, including postponement of childbearing, early menarche, genetic mutations, and most importantly, physical inactivity and obesity [2]. Despite the fact that breast cancer has a good prognosis if discovered at early stages, 50-80% of the cases are unfortunately discovered in later stages, making the tumor cells more resistant to therapy, hence favoring a quiet poor prognosis [3].

A number of factors influence management plans and decision-making for patients with breast cancer. These factors include tumor morphology, grade, size, metastases, and notably the expression of estrogen receptors (ER), progesterone receptors (PR), and human epidermal growth factor receptor 2 (HER2) [4–6]. Primarily, several biological subtypes of breast cancer exist and can be delineated based upon their genotypic and phenotypic features. This classification is achieved by a multitude of laboratory techniques including immunohistochemistry and genetic expression profiling. Further genetic profiling and molecular analysis of breast cancer led to its classification into several subtypes including ER+ luminal A and luminal B, HER2-enriched, and triple-negative breast cancer [7]. Histologic analysis of breast cancer is diagnostic and stratifies breast cancer to its subtypes. It was identified that the most common subtype is invasive ductal carcinoma, which makes up 50%-75% of patients. The second most common subtype is invasive lobular carcinoma, making up 5%-15% of patients, while mixed ductal/lobular carcinomas and other rarer histological subtypes make up the remainder of patients [8].

Interestingly, according to a cohort study conducted in the UK, a higher body mass index (BMI) in postmenopausal women was associated with a 20-40% increased risk of breast cancer development. This led to the development of a concept known as the “obesity paradox” which implies that while morbidly high BMI was associated with a poorer prognosis, a moderately high BMI showed a better prognosis and response to therapy in premenopausal women specifically [9, 10].

An overview of the breast tumor microenvironment (TME) will help provide a better understanding of the role of a variety of cell types, their effects on each other, and the surrounding cells leading to the proliferation and metastasis of the tumor. There are various cell types in the breast cancer TME such as breast cancer cells, epithelial mesenchymal cells (EMTs), and stromal cells which include fibroblasts, adipocytes, endothelial cells, and immune cells. Cell adhesion is reduced between tumor cells in the TME in comparison to normal epithelium whereby cells are tightly attached to each other via cell junctions and cell adhesion molecules (CAMs). This feature of decreased adherence facilitates its dissociation and proliferation [11]. Tumor cells divide in an uncontrollable manner, which mandates different oxygen and nutrient supplies, needs to the cancerous cells at different locations resulting in a hypoxic environment that alters protein expression leading to further mutations in tumor cells and triggers angiogenesis allowing direct access to blood and lymphatic fluid for metastasis [12]. Hence, cellular mutations could result in uncontrollable cell division and hence the development of a tumor [13].

One of the cell types present in the tumor microenvironment is fibroblasts. Upon tissue insult, fibroblasts are converted into myofibroblasts that could further transform into tumor-associated fibroblasts (TAFs). TAFs play a major role in promoting tissue fibrosis, angiogenesis, immunosuppression, and metastasis. Additionally, cytokines in the TME facilitate the conversion of adipocytes into tumor-associated adipocytes (TAAs). TAAs secrete additional cytokines, adipokines, free fatty acids (FFAs), and matrix metalloproteinases (MMPs), enrolling immune cells to the TME and leading to further inflammation. Other cell types include vascular cells that constitute a monolayer of endothelial cells (ECs) that are in direct contact with the blood. ECs direct inflammatory cells towards foreign molecules and the inflammatory milieu in response to infection or inflammation [14]. Finally, immune cells are recruited to the TME, as it represents a highly inflammatory site [15, 16].

## 2. Natural Killer Cells

The immune system is implied in the defense against pathogens and tumor cells. Both branches of the immune system, namely, the innate and adaptive immune systems with their cellular components, soluble molecules, and cellular receptors that have different functions, are ultimately aimed at eradicating pathogens or tumor cells from the body. The innate immune system constitutes the initial and early response of the body and thus acts rapidly and in a nonspecific manner to prevent the spread of the foreign pathogen. This is achieved by a plethora of factors including but not limited to complement activation and cytotoxic molecule release as well as activation of other immune cells [17].

Natural killer cells (NK cells) are large granular lymphocytes that represent a crucial constituent of the innate immune system as they mediate immunity against viruses, bacteria, parasites, and most importantly, tumor cells [18]. NK cells are defined by the expression of an adhesion molecule CD56 and by the absence of the T cell marker CD3 [19]. Several subdivisions of NK cells exist based on the cellular expression of CD56 and the Fc (gamma) receptor CD16. The most common subdivision of NK cells is based on function, whether they primarily induce cytotoxic activity or release proinflammatory cytokines. The cytotoxic cells are mostly CD56^dim^ CD16^bright^ and represent about 90% of all NK cells [20]. Cytotoxicity is mediated against target cells by the secretion of cytotoxic molecules or death receptor-mediated apoptosis. Granzymes and perforins are cytotoxic molecules that result in cell death. Another possible method is via activation of death cell receptors such as Fas ligand and TNF-related apoptosis-inducing ligand (TRAIL), which leads to the classical caspase-mediated apoptosis. The other subtype includes CD56^bright^ CD16^dim^ that has an immunoregulatory role and releases many cytokines such as IFN-*γ* and TNF-*α* [21–23].

In addition, NK cell functionality is dependent and dismantled by several factors. For instance, aging is a condition that greatly impairs NK cells' function [24]. Another major factor that will be further discussed in this review is obesity. Several factors are believed to affect NK cell function in obese patients in comparison to lean patients. This formulates a triangle of interest: obesity, breast cancer, and NK cell dysfunction. In this review, we aim to further expand the link between these factors and their effect on the innate immune system in fighting breast cancer.

### 2.1. Mechanism of Natural Killer Cell Activation

NK cell function is tightly regulated by a repertoire of membrane-expressed inhibitory and activating receptors, which are the “nuts and bolts” of NK cell function [25]. Inhibitory receptors recognize normal healthy cells via the self-major histocompatibility complex (MHC) class I molecules and thus play a crucial role in self-tolerance. NK cell inhibitory receptors include members of the C-type lectin-like receptor, leukocyte immunoglobulin-like receptors (LILRs), and the killer immunoglobulin-like receptors (KIRs) [26, 27]. NK cells are activated when they encounter other cells that are not expressing MHC class I receptors like cancer cells or viral-infected cells. Such cells are undergoing stress which downregulates the expression of MHC class I and upregulates the expression of other molecules/ligands that further activate the NK cells. Upon this interaction, NK cells either are either directly activated and eliminate the cells by cytotoxicity or indirectly release their proinflammatory cytokines which will eventually result in the death of these stressed cells. The lack of MHC class I expression is insufficient for the activation of NK cells, but other NK receptors contribute to the complete activation of NK cells when they are stimulated, including NKG2D and NCRs like NKp30,NKp46, and NKp44 [28].

### 2.2. Natural Killer Cells and Breast Cancer

Being innate cells, NK cells can lyse tumor target cells without prior sensitization or clonal expansion, unlike T cells. NK cells play a fundamental role in cancer immunosurveillance by performing their antitumor activity [29, 30]. This has been supported by studies where elimination of NK cells led to increased malignancy occurrence [31–33]. NK cells perform antitumor activity when the expression of MHC class I molecules is downregulated. Additionally, upregulation of stress-induced molecules such as ligands of the activating receptor C lectin receptor D (NKG2D) on tumor cells marks them susceptible to NK cell killing [34]. Moreover, NK cells have been found to enhance T cell infiltration, thus triggering immune responses through their cytokines and chemokine secretion [35, 36]. In addition, NK cells possess antimetastatic activity by possible elimination of circulating tumor cells [36, 37].

The exact mechanism of how the innate immune system and specifically NK cells interact with breast cancer cells is complex and is yet to be understood. However, several factors that are released by breast cancer cells and constitute the TME are believed to contribute to this complex physiologic reaction to tumor cells. Additionally, the TME is thought to play a major role in various processes such as tumor development, progression, growth, and metastasis. Most importantly, TME mediates immune suppression leading to tumor progression by inhibiting the immune system antitumor activities [38, 39].

### 2.3. Role of Natural Killer Cells in Tumor Microenvironment

The protective functions of NK cells are hindered by the suppressive cytokines found in the TME. Among the cytokines released in the TME is tumor growth factor- (TGF-) *β*, which is released by the tumor cells, Tregs, and other stroma cells. TGF-*β* is responsible for inhibiting the function of NK cells in both direct and indirect manners. Initially, TGF-*β* suppresses the IFN-*γ* production along with the levels of NKG2D and NKp30 on the cell surface. Moreover, TGF-*β* binds to certain receptors that contain TGFBR1 and TGFBR2 subunits that will propagate the signal transduction of phosphorylating SMAD2 and SMAD3 proteins which will bind to SMAD4 resulting in a heterotrimeric transcriptional structure. The SMAD proteins are the main signal transducers for the receptors of the TGF-*β* superfamily. The role of TGF-*β* was not only limited to disabling NK cells but also to inducing the conversion of NK cells into NK-ILC-1, intermediate cell type (int ILC1s) which is by default a weaker cytolytic cell compared to NK cells. This will end up with a poorer cancer surveillance and eventually pave the way for further cancer evasion [40].

Metabolic derangement is one of the hallmarks characterizing the TME, leading to NK cell dysfunction. For instance, lactate level is increased in the TME and leads to suppression of cytotoxic T cells and NK cell proliferation and reduction of their cytokine production [41]. Furthermore, hypoxic environment such as that in the TME downgrades the NK cell functions by inhibiting the activating receptors such as NKG2D, NKp30, and CD16. Additionally, studies demonstrated that the depletion of NK cells prior to implantation of tumor cells in mice was found to be associated with a more aggressive picture of tumor metastasis [42–44].

An emerging role of NK cells in targeting tumor cells is being recognized by their action on cancer stem cells. Cancer stem cells (CSCs) are undifferenced cells involved in the growth of tumors. They are characterized by an expression profile consisting of low levels of CD54 and PD-1 and high expression of CD44. This profile increases the susceptibility of CSC to be targeted by NK cells but conversely induces their resistance to chemotherapy. NK cells drive the CSCs to differentiate in a way where the expression of MHC-1, CD54, and PD-L1 is elevated, resulting in stunted tumor growth and decreased metastasis. This delineates the importance of NK cells not only in restraining tumors but also in limiting their growth. On the contrary, other studies demonstrated that the CSCs in breast tumors are resistant to any NK cell activity and thus, more research is needed to further elucidate and establish the true link between NK cells and CSCs of breast cancer [45].

## 3. Obesity

Obesity can be defined as the excessive and abnormal accumulation of adipose tissue and is commonly classified based on the body mass index (BMI). The BMI of an individual can be calculated by dividing the body weight in kilograms by the height in meters squared (kg/m^2^) [46, 47].

Obesity has come to light as a major public health problem that leads to approximately 4 million deaths and 120 million disability-adjusted life-years (DALY). According to the WHO, obesity has tripled on a global scale since 1975. In 2016, 1.9 billion people at 18 or more were overweight or obese. Unluckily, obesity rates in the MENA region are not updated, but the indices show that the number of obese people is escalating. This could be attributed to the higher levels of urbanization and technical advancements in the MENA region that contribute to a sedentary lifestyle and unhealthy food options [48]. Most of the subsequent complications caused by obesity share a common feature which is a state of subclinical chronic inflammation that is a crucial component of tumor development and progression [49].

Being obese or overweight is a well-known risk factor for the development of several chronic health disorders such as type 2 diabetes and cardiovascular diseases, among many others. Along with obesity comes an increased susceptibility to infection and decreased ability to fight off infections efficiently. Most importantly, there is an increase in the incidence of several types of cancers, e.g., colorectal, endometrial, pancreatic, and breast cancer, with an increased incidence and poorer prognosis in obese patients. In fact, 14-20% of the cancers have been attributed to obesity [50].

Consequently, obesity results in the increase in the accumulation of adipose tissue mass. Adipose tissue, in lean state, acts as an energy-storage reservoir and the largest endocrine organ that is thought to secrete a multitude of adipokines including but not limited to leptin, adiponectin, resistin, and estrogens as well as interleukin-6 (IL-6), all of which orchestrate a variety of reactions in the body. On the contrary, examples of anti-inflammatory and adipose-resident immune cells are Tregs, eosinophils, T-helper 2 cells, and M2 macrophages [51]. Adipocytes are the prominent producers of leptin in the body, where leptin acts a stimulator of multiple proinflammatory reactions and the production of IL-1, IL-6, IL-12, TNF-*α*, COX2, and nitric oxide (NO). Leptin levels are found to be higher in obese patients in comparison with lean patients contributing to the chronic inflammation that occurs in obesity. In fact, a study suggested that levels of leptin could be used to predict type, grade, prognosis, and recurrence in breast cancer based on its immunohistochemical staining [15, 52]. It is worth mentioning that the expansion of adipocytes to meet the increased energy storage demands could eventually cause these cells to become apoptotic, thus attracting proinflammatory macrophages and forming crown-like structures: a hallmark of the inflammatory environment in adipose tissue [53].

### 3.1. Breast Cancer and Obesity

Even though inherited genetic factors such as BRCA1/2 mutations result in 5-10% of cases of breast cancer, lifestyle is now considered as an increasingly contributing factor to the etiology of breast cancer [54]. The incidence of breast cancer recurrence and mortality rate increases with obesity due to the dysregulation of a variety of biological and nonbiological factors. These factors include advanced stages of breast cancer presentation, increased risk of second primary cancer (i.e., primary cancer in other tissues), and the use of a suboptimal level of chemotherapeutic agents compared to the relative body size [55].

There is an aberration involving multiple molecular pathways involving adipokines, endogenous sexual hormonal levels, and most importantly, inflammation [56]. Out of these molecular pathways, there is an abnormal regulation in the levels of estrogen due to the aromatization of the adipose tissue. Inflammatory cytokines are thus recruited such as TNF-*α*, IL-6, and prostaglandin E2 adipokines, not to mention oxidative stress, which contributes to carcinogenesis. The molecular factors induce intracellular interference which activate mitogen protein kinase (MAPK) and phosphatidylilinositol-3-phosphate/mammalian target of rapamycin (mTOR pathway), which play a role in the progression of cell cycle and protein synthesis. In breast cancer, the associated genes of obesity collectively lead to an increase in fat mass and production of cytokines such as leptin, which has been associated with a higher risk of cancer development [56].

In addition, the association of breast cancer with adiposity has been linked to higher energy states that may enhance tumor growth by providing an increased level of ATP for inducing cell growth and replication. There is evidence for a “metabolic threshold” in promoting breast cancer, which was supported by the development of targeted metabolic inhibitors as cancer therapeutics [57, 58].

### 3.2. Natural Killer Cells and Obesity

There is evidence that points towards a decrease in the number of NK cells in the blood and tissues of obese individuals. Furthermore, there is a decrease in the number of NK cells in the blood and organs of obese rats [59–62]. Other data reveal no change in the number of NK cells or even an increase in the NK cells present in the blood and tissues of obese individuals [60–66]. This can be attributed to several various factors including but not limited to metabolic differences between species and strains as well as discrepancies between the development and migration processes of NK cells in different species [67]. In addition, such discrepancies could be due to several reasons including the choice of markers and quantitative methods used for NK cells as well as differences in the study population such as BMI, gender, ethnicity, body composition, and variance [68, 69].

From a functional prospective, data in obese individuals revealed a clear decrease in the activating receptors on NK cells, namely, NKp46, as well as TRAIL, functional markers of NK cells [60]. On the other hand, other studies revealed a highly activated status of NK cells in obese individuals, as highlighted by an increase in the expression of CD69 and NKp46 as well as PD-1, while there was a decline in the inhibiting complex NKG2A/CD94, thus indicating an activated status of NK cells [70]. While NK cells seemed to be increasingly activated, their functionality was greatly impaired as evidenced by a decreased secretion of mediators necessarily for their function such as granzyme B, perforin, and macrophage inflammatory protein *β*. This decline in functionality could be attributed to exhaustion of NK cells which occurs faster in obese individuals compared to normal-weight individuals [71, 72]. To further confirm findings on the effect of obesity on NK cell function, some studies interestingly demonstrated that the impaired NK cell function can be restored and normalized following the loss of body weight and fat mass in obese individuals. This includes an increase in the CD69 levels and granzyme B secretion as well as a decrease in IFN-*γ* production [73]. On the other hand, several studies associated the caloric restriction with improved NK cell cytotoxicity and elevated expression of activation markers CD69, TNF-*α*, and GM-CSF. However, few studies showed a decrease in NK cell numbers and cytotoxicity upon weight reduction [59].

Interestingly, chronic low-grade inflammation of fat tissue can be appreciated by an increase in immune cells such as macrophages, T cells, and NK cells [74]. Studies show varying data with regard to NK cells in adipose tissue. Some studies reported an increase in NK cells in adipose tissue of obese individuals, while others demonstrate a decreased or no change in the number of NK cells between obese and lean individuals [74–78]. However, a shift from the cytotoxic CD56^dim^ subpopulation of NK cells to the CD56^bright^ cytokine secreting subset was observed in obese individuals, which serves to explain the reason for increased cytokine secretion in obese individuals [79]. In mice, an increased level of activating receptor NKp46 on adipose tissues seemed to increase the proliferation of NK cells that secrete IFN-*γ*, which eventually leads to a polarization in macrophages to the M1 proinflammatory macrophages [74]. A decreased expression of NK cells activating receptors such as NKp30 and NKp44 was observed in adipose tissue of obese individuals, which might be a contributing factor to the increased risk of cancer development in obese individuals as well as increased susceptibility to infection [79].

In obesity, there are higher levels of free fatty acids (FFAs) circulating freely all over the body. NK cells have shown to absorb these FFAs and accumulate lipid droplets. Interestingly, NK cells that accumulated more lipid droplets had merely zero perforin and granzyme levels as detected by flow cytometry, resulting in impaired NK cell cytotoxicity against cancer cells and hence helping the tumor cells to further grow and metastasize. Lipid-rich environment was proven to disturb the mTORC1 pathway in NK cells, a pathway that plays a prominent role in the NK cell function and IFN-*γ* production [80]. On the other hand, obesity could be a stimulator of the peroxisome proliferator-activated receptor PPAR*α*/*δ* target genes in NK cells that encourage the NK cells to further accumulate more lipids and hence hinder the cytotoxicity of NK cells [81]. NK cells residing in the visceral adipose tissue (VAT) are activated due to the increased stimulation of NCR-1 signaling by the surrounding adipocytes. As a result, IFN-*γ* production is elevated which elicits M1 polarization in the macrophages residing in the adipose tissue. Moreover, M1 macrophages have a pivotal role in inducing further inflammation, further contributing to insulin resistance and obesity. This was further proved by a study where the total number of macrophages and specifically M1 macrophages was lower in NK-depleted mice compared to normal mice fed with a high-fat diet (HFD) [74].

Moreover, NK cell functions and cytotoxicity were seen to improve following restriction of energy intake and low-fat diet [82–84]. Most studies demonstrate a stimulating effect of caloric restriction on NK cell function, while others have demonstrated opposite findings with reduced killing and impaired maturation of NK cells as well as decreased cytokine production [85, 86]. Lack of weight cycling was associated with higher NK cell activity, demonstrating improved NK cell function with lack of weight gain and weight loss [87]. Similar findings were seen with bariatric surgery effects with contradicting findings [88, 89].

Another possible link between NK cells, obesity, and cancer was highlighted in the study by Mariani et al. This study showed a reduced NK cell number in the colon tissue of obese patients in comparison to normal-weight patients. Hence, this could be a contributing factor to the increased risk of colon cancer in obese individuals [84].

## 4. Natural Killer Cell-Mediated Immunotherapeutics in Breast Cancer

As noted from the previous sections, NK cells are unique in exerting an innate immune activity that is antigen independent. They represent an excellent companion to immunotherapy in clinical settings by having the ability to distinguish “self” from “missing-self.” Over the last few years, many studies showed NK cells as promising effectors in tumor therapy [90]. There is mounting evidence of the potential use of NK cells as a therapeutic tool in clinical practice.

There are still many open research questions related to NK cell metabolism modification in a trial to enhance the survival and activity of those key promising cells in the TME of solid tumors, including breast cancer. Several metabolic modulation strategies were investigated to support the NK cell survival and activity in the TME. Naturally, NK cells survive for 2 weeks [18, 20], but the infusion of IL-2 and/or IL-15 showed advantageous effects on the NK cell survival (as in the context of adoptive transfer therapy in patients with acute myeloid leukemia) [91]. Another study by Liu et al. showed that IL-15 production by transduced cord blood NK cells critically improved their function [92]. These strategies are likely to improve delivering this therapy in the clinical setting and to overcome a major limitation to current CAR-T cell therapies. It should be noted that defining a metabolic pathway for the NK cells is a step towards identifying a therapeutic target that addresses this pathway and specifically activates the cytotoxic activity of the NK cells. Production of certain metabolites limits the survival and function of NK cells, including pyruvate dehydrogenase kinase 1, lactate dehydrogenase A (LDHA), and adenosine (ADO) [93]. Interestingly, lipid-lowering drugs represent a potential therapy for patients with ER-positive breast cancer. In contrast, a recent study by Qin et al. on liver-tumor-bearing murine model suggested that cholesterol accumulation in NK cells enhances their antitumor ability through increasing the formation of lipid rafts [94]. The results of the latter study reflect the diverse function of lipid metabolism in different cancers. In addition, direct activation of the citrate-malate shuttle was demonstrated to enhance glucose metabolism and hence NK cell cytotoxicity and may have a role in their persistence/survival [95]. Additionally, a study on CD8+ T cells reported that cell activation under the effect of 2-deoxy-glucose (2DG) inhibitor enhanced the generation of memory cells and antitumor functionality, which could be applied to NK cells as well [96].

On the other hand, activation of the transcription factor SREBP (sterol regulatory element-binding protein) and its control of glucose and lipid metabolism were found to be essential for the function of NK cells. Furthermore, SREBP was reported to be essential for activated NK cells, as it provides metabolic reprogramming [97]. A study by Wu et al. showed that SREBP inhibitors such as 27-hydroxycholesterol (27HC), accumulating in the TME, partly affect SREBP-related glycolysis in ER-positive BC [98]. Interestingly, Baek et al. reported that treating mice submitted to high-cholesterol feed with an inhibitor of CYP27A1, an enzyme important in 27HC biosynthesis, clearly decreases the number of metastases in mice and reverses the immune suppressive environment [99].

## 5. The Triad: Effect of Obesity on Natural Killer Cell Functions in Breast Cancer

**Figure 1:**
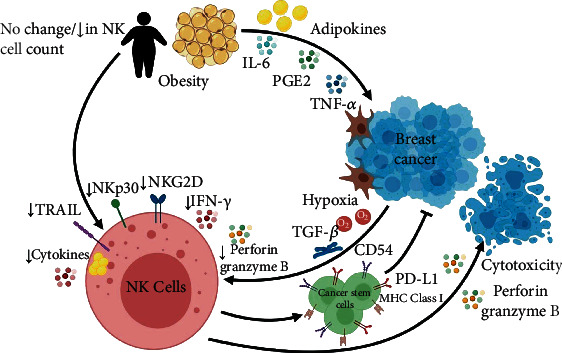
Triad of obesity, natural killer cells, and breast cancer. Typically, natural killer (NK) cells release perforin and granzyme B, molecules that cause cytotoxicity and induce apoptosis of breast cancer (BC) cells. Additionally, NK cells stimulate the expression of CD54, PD-L1, and MHC class I molecules on cancer stem cells, which inhibit the metastasis and proliferation of breast cancer cells. In turn, hypoxic environment of breast cancer cells and secreted TGF-*β* by stromal cells in the tumor microenvironment (TME) cause a reduction in the activating receptors (NKG2D and NKp30) as well as the NK cell function: IFN-*γ* production and cytotoxicity via perforin and granzyme B release. Similarly, adipose tissue causes a reduction in TRAIL, NKp30, and NKG2D expression in obese patients. Lipid droplet accumulation in NK cells leads to a reduction in cytokine release such as IFN-*γ*. Controversial data were reported regarding the count and status of NK cells in obese individuals. On the other arm, adipose tissue secretes IL-6, PGE2, TNF-*α*, and adipokines such as leptin which trigger the proliferation of breast cancer cells via the MAPK and mTOR pathways.

Glucose and lipid metabolism is generally highly activated in breast cancer. This was demonstrated by several metabolomic studies of breast cancer specifically revealing enhanced fatty acid synthase and glycolysis. Obesity exerts a status of NK cell immune paralysis by affecting the NK cell metabolism and trafficking in the TME [81]. Previous reports showed that metabolic reprogramming of NK cells in obesity limits the antitumor responses through different mechanisms, mainly through PPAR*α*/*δ* pathway and inhibition of mTOR-mediated glycolysis [81]. Michelet et al. showed a plausible mechanistic effect of obesity that enhances lipid accumulation in NK cells through a peroxisome proliferator-activated receptor (PPAR), leading to inhibition of the mechanistic target of rapamycin- (mTOR-) mediated glycolysis in NK cells. In obesity, PPAR*α*/*δ* target genes are highly upregulated, thus causing inhibition of IFN-*γ* production as well as the downstream transcription of other cytotoxic granules in adipose tissue NK cells [81]. This illustrates the inhibitory effect of obesity on NK cell function, which in turn contributes to further growth and metastasis of tumor cells. It is important to note, however, that NK cell function can also be impeded by breast cancer cells through the aforementioned factors such as hypoxia and increased production of TGF-*β* which lead to decreased perforin and granzyme B secretion from NK cells, hence reducing its antitumor activity (Figure 1). Collectively, both breast cancer and obesity lead to enhanced breast cancer proliferation through distinct but directly related pathways, thus promoting tumor growth and metastasis.

## 6. Conclusions

In this review, we aimed to investigate the triad: obesity, breast cancer, and NK cells, to aid in the understanding of the various effects of these players in breast cancer development and progression. Extensive research is still ongoing to pin down the biomarkers associated with various types of breast cancer that impede NK cell function. Targeting these factors will help in designing targeted immunotherapy with a low side-effect profile [100]. Despite the fact that NK cells are short-lived and targeting them might not help in having a prolonged anti-inflammatory reaction against tumor cells, ongoing research is still trying to obtain features of immunological memory which result in increased NK cell survival and efficacy [101, 102]. More studies are required to solidify the link between obesity, breast cancer, and NK cells and to identify other factors that may play a role in their interaction.

## Abbreviations

27HC: 27-Hydroxycholesterol
2DG: 2-Deoxy-glucose
ADO: Adenosine
BMI: Body mass index
CAMs: Cell adhesion molecules
DALY: Disability-adjusted life-years
ECs: Endothelial cells
EMTs: Epithelial mesenchymal cells
ER: Estrogen receptors
FFAs: Free fatty acids
HER2: Epidermal growth factor receptor 2
HFD: High-fat diet
KIRs: Killer immunoglobulin-like receptors
LDHA: Lactate dehydrogenase A
LILRs: Leukocyte immunoglobulin-like receptors
MAPK: Mitogen-protein kinase
MMPs: Matrix metalloproteinases
mTOR: Phosphatidylilinositol-3-phosphate/mammalian target of rapamycin
NK cells: Natural killer cells
NKG2D: C-type lectin receptor D
NO: Nitric oxide
PPAR: Peroxisome proliferator-activated receptor
PR: Progesterone receptor
SREBP: Sterol regulatory element-binding protein
TAA: Tumor-associated adipocytes
TGF: Tumor growth factor
TME: Tumor microenvironment
TNF: Tumor necrosis factor
TRAIL: TNF-related apoptosis-inducing ligand
VAT: Visceral adipose tissue.
